# Whole-genome sequencing of Tarim red deer (*Cervus elaphus yarkandensis*) reveals demographic history and adaptations to an arid-desert environment

**DOI:** 10.1186/s12983-020-00379-5

**Published:** 2020-10-16

**Authors:** Buweihailiqiemu Ababaikeri, Shamshidin Abduriyim, Yilamujiang Tohetahong, Tayerjan Mamat, Adil Ahmat, Mahmut Halik

**Affiliations:** 1 College of Life Sciences and Technology, Xinjiang University, Urumqi, 830046 Xinjiang China; 2 College of Xinjiang Uyghur Medicine, Hoten, 848000 Xinjiang China; 3 College of Life Science, Shihezi University, Shihezi, 832003 Xinjiang China; 4 Department of Ecology, Hubei Key Laboratory of Cell Homeostasis, College of Life Science, Wuhan University, Wuhan, 430072 Hubei China

**Keywords:** Arid-desert environment, *Cervus elaphus*, Environmental adaptability, Population demographic history, Tarim red deer, Whole genome sequencing

## Abstract

**Background:**

The initiation of desert conditions in the Tarim Basin in China since the late Miocene has led to the significant genetic structuring of local organisms. Tarim Red Deer (*Cervus elaphus yarkandensis*, TRD) have adapted to the harsh environmental conditions in this basin, including high solar radiation and temperature, aridity, and poor nutritional conditions. However, the underlying genetic basis of this adaptation is poorly understood.

**Results:**

We sequenced the whole genomes of 13 TRD individuals, conducted comparative genomic analyses, and estimated demographic fluctuation. The ∂a∂i model estimated that the TRD and Tule elk (*Cervus canadensis nannodes*) populations diverged approximately 0.98 Mya. Analyses revealed a substantial influence of the Earth’s climate on the effective population size of TRD, associated with glacial advances and retreat, and human activities likely underlie a recent serious decline in population. A marked bottleneck may have profoundly affected the genetic diversity of TRD populations. We detected a set of candidate genes, pathways, and GO categories related to oxidative stress, water reabsorption, immune regulation, energy metabolism, eye protection, heat stress, respiratory system adaptation, prevention of high blood pressure, and DNA damage and repair that may directly or indirectly be involved in the adaptation of TRD to an arid-desert environment.

**Conclusions:**

Our analyses highlight the role of historical global climates in the population dynamics of TRD. In light of ongoing global warming and the increasing incidence of droughts, our study offers insights into the genomic adaptations of animals, especially TRD, to extreme arid-desert environments and provides a valuable resource for future research on conservation design and biological adaptations to environmental change.

## Background

Adaptation to diverse and changing environments is a fundamental principle in evolutionary biology, and estimating the potential for adaptive evolution is critical when identifying populations/species at risk of extinction due to environmental change [1]. In wild animals, climate-induced extinctions, distributional and phenological changes, and species’ range shifts have been documented at an increasing rate; in particular, the influence of climate change on organisms is exacerbated in extreme environments, such as on plateaus and in desert regions [2]. Adaptation is a complex process that involves many biological systems and quantitative trait loci, each having a small but cumulative effect on the overall expression of the phenotype [3–5]. Identifying genes that are under natural selection and understanding how species have adapted genetically to extreme environments provide information for design of conservation programs to effectively protect important species under scenarios of changing climate [6]. Revealing how populations/species adapt to changing environments thus plays a critically important role in assessing their evolutionary and ecological dynamics, and predicting population resilience to climate change [7].

**Fig. 1:**
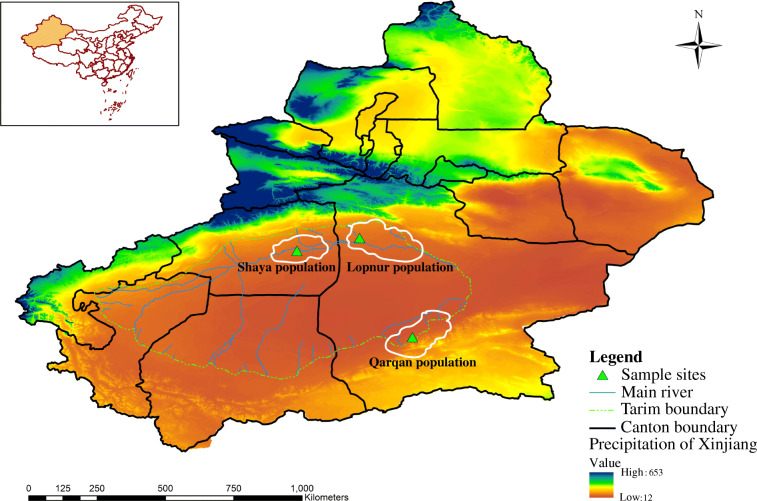
Distributional map of Tarim red deer in Xinjiang, China. The map shows the distribution of TRD populations (areas marked with white lines) and the origin of samples used in this study, and depicts annual average precipitation in the study area

Red deer (genus *Cervus*) are widely distributed around most of Holarctic region, from northern Africa, southern Europe (Spain, Sardinia, Corsica) and western Europe (Scotland) across Asia to North America. Species in the genus show a broad range of morphological features (i.e. antler and body size; coat colour) in different climatic conditions, and show considerable biological adaptations for survival under diverse environmental conditions—for example, extreme cold, hot, or moderate temperatures; extremely dry or wet conditions; high altitude; steppe-like habitats, forests, and swampy river plains [8]. The Tarim red deer (TRD; *C. e. yarkandensis*) is a subspecies of *Cervus elaphus* mainly distributed along the Tarim River and its tributaries in the Tarim Basin in Xinjiang Uygur Autonomous Region (XUAR), China (Fig. 1). Aridification and desertification in the basin date back to 5.3 Mya (millions of years ago [9, 10];). Honored as “the flower of Asia” in the international market owing to its large quantities of velvet antler with good quality [11, 12], and being the main large-sized ungulate that plays an important role in maintaining arid and semi-arid forest ecosystems and contributing biodiversity, the Tarim red deer is economically and ecologically the most important animal in Tarim Basin, and has been designated as a Category II protected animal in China [13]. In a long evolutionary process, they have been subjected to strong environmental pressures. The basin is extremely arid, with the average annual precipitation less than 10 mm and the average annual evaporation reaching 1900–2700 mm [14]. There is intense solar radiation and heat; the annual average temperature in the distributional area is 10.5 °C, with temperatures up to 40 °C in summer and surface temperatures reaching over 60 °C [13, 15, 16]. Feeding conditions are poor; TRD forage mainly on *Phragmites communis*, *Tamarix ramosissima*, and *Populus diversifolia*, which are difficult to chew [17], and drink highly mineralized water [13]. Several studies have focused on the adaptive evolution of TRD in this arid-desert environment (e.g. [13, 18–20]), but the mechanisms underlying adaptive traits are poorly understood.

Advances in next-generation sequencing technology now make it possible to sequence individuals’ whole genomes and detect selective sweeps, which can provide insights into genome biology and the mechanisms of adaptation to extreme environments. Sequencing cost and time have dramatically decreased [21], allowing whole genome sequencing (WGS) data to be obtained for many individuals, allowing variation to be examined among data sets [22]. Genomic differences can shed light on the genetic basis of adaptation to diverse environments and provide insights into functionally important genetic variants [23]. It is now possible to combine WGS data and population genomic approaches to characterize adaptive variation in an unprecedented way. The integration of genomic information will undoubtedly lead to better management and sustainable utilization of genetic resources.

In recent years, WGS has been widely used to explore adaptive genetic variations of humans and other species living in harsh or extreme environments, for instance, goat (*Capra hircus*) and sheep (*Ovis aries*) in hot, arid environments [3, 24–26]; domestic Bactrian camel (*Camelus actrianus*) in severe desert conditions [27, 28]; and Tibetan yak (*Bos grummiens*), Tibetan antelope (*Pantholops hodgsonii*), Tibetan mastiff (*Canis lupus familiaris*), and Tibetan sheep (*Ovis aries*) at high altitudes on the Tibetan plateau [29–33].

Here, we Illumina-sequenced the whole genomes of multiple TRD, compared them to previously published Tule elk (*Cervus canadensis nannodes*) genomic data [34], revealed the divergence times and demographic history of these species, as well as identified a set of candidate genes and genomic regions under direct or indirect selection in an extremely arid environment. Our findings provide a more complete picture of TRD evolutionary history, making it possible to investigate features unique to this species and how these might reflect adaptation to a hot, arid environment, all of which are imperative for a better understanding of possible responses to current and future climatic changes. Our results are important for their potential application to functional genomics and in the design of programs to protect genetic resources in view of ongoing global warming and the increasing incidence of droughts.

## Materials and methods

### Sampling

Samples were collected from 13 TRD for sequencing. Muscle samples from three free-ranging individuals that had been poached or died of natural causes were provided by the Local Forestry Bureaus of Qarqan (two individuals) and Shaya (one individual) counties in XUAR, China. Blood samples were obtained in 2018 from 10 captive individuals by means of jugular venipuncture into EDTA-coated vacutainer tubes; these individuals were from a farmed population in Lopnur, XUAR, that had undergone no known hybridization with any other red deer lineages (Fig. 1, Additional file 1: Table S1). Blood collection was conducted in strict accordance with the Animal Ethics Procedures and Guidelines of the People’s Republic of China.

The domestication of TRD in Xinjiang began in 1958 with the capture of about 2000 wild individuals, mainly juveniles, from their natural habitat [35]. The capture of wild individuals continued until the TRD was listed as a National class II key protected species in 1982 and capture became prohibited [35]. The average mutation rate per base per generation (μ) for a similar species, Milu (Père David’ s deer, *Elaphurus davidianus*), was found to be 1.5 × 10^− 8^ [5], and the generation time (g) of red deer is 6.3 years [36]. Thus, the impact of 36 years of captive history has likely had a negligible effect on this species’ genome. Importantly, the blood samples we used in this study were collected from farms in Lopnur County, with a dry climate typical of temperate continental plains (average annual precipitation 45.2 mm).

Tule elk (*Cervus canadensis nannodes*) are typified by having lower body masses, lighter pelage, and the longest tooth rows of any North American subspecies [37]. They are far more widespread in North American, occupying much of California’s extensive lower elevation oak woodland and have unique morphological adaptation to mild Mediterranean grasslands [38, 39]. They are sensitive to variation in climate, especially precipitation [38], and a hot, dry climate, or droughts, appear to have had substantial negative effects on elk herds independent of hunting pressure [40]. For these reasons, previously published genomic data of four Tule elk that were sampled from four geographically distinct populations across northern California [34] with average annual precipitation more than 324 mm were retrieved from NCBI (BioProject ID PRJNA345218) to represent deer from a non-arid-desert habitat for comparison.

### Sequencing, read mapping, and quality control

The whole genome of each of the 13 TRD samples was Illumina-sequenced. For each sample, a library was constructed with an insert size of 350 bp from high-quality DNA and then sequenced on an Illumina Hiseq Xten platform using paired-end (PE) 150 bp reads following standard procedures. The average sequencing depth was ~ 8.57X per sample. Adapter and low-quality read pairs, which included reads with > 10% unidentified nucleotides (nt) and reads containing > 50% low-quality bases (Q < 5) in paired reads, > 10 nt aligned to the adaptor, allowing ≤10% mismatches, were filtered from the sequenced reads (raw reads) to obtain high-quality reads (clean reads). High-quality reads were then mapped to the CerEla1.0 red deer (*Cervus elaphus hippelaphus*) reference genome [41] available from NCBI, using Burrows-Wheeler Aligner software Version: 0.7.8 [42] under the parameters ‘mem -t 4 -k 32 -M.’ To reduce mismatches generated by PCR amplification before sequencing, duplicate reads were removed by SAMtools Version: 0.1.19 [43] with the parameter ‘-type rmdup.’

The genome was divided into segments and analyzed in parallel. After alignment, we performed SNP calling on a population scale by using a Bayesian approach implemented in the package SAMtools v0.1.19.We then calculated genotype likelihoods from reads for each individual at each genomic location, and the allele frequencies in the sample with a Bayesian approach. To identify SNPs, we used the ‘mpileup’ command and parameters ‘-q 1 -C 50 -t SP -t DP -m 2 -F 0.002.’ (See Additional file 2: Table S2 for parameter illustration). High-quality variants were selected among raw SNPs based on the following quality scores: coverage depth ≥ 3, RMS (root mean square) mapping quality ≥20 (i.e. SNPs with a sequencing error rate > 1% were filtered out); miss ratio ≤ 0.2; and MAF (minimum allele frequency) ≥ 0.01. If two SNPs were less than 5 bp apart, both were removed.

### Positional annotation of genetic variants

SNPs were annotated against the CerEla1.0 genome (CerEla1.0.gff file) by using the package ANNOVAR version 2013-05-20 [44]. Based on the genomic annotation, SNPs were categorized as occurring in exons, in introns, at splice sites (within 2 bp of a splice junction), upstream or downstream regions (within 1 kb upstream or downstream from a transcription start site) and intergenic regions. SNPs in exons were further categorized as synonymous (caused no amino acid change) or nonsynonymous (caused an amino acid change, or caused the gain or loss of a stop codon).

### Linkage-disequilibrium analyses

To assess the linkage disequilibrium (LD) pattern between the TRD and Tule elk populations, the coefficient of determination between any two loci in each population was calculated by using PopLDdecay Version 3.4 [45] under the parameters ‘-MaxDist 500 -MAF 0.05 -Miss 0.1 -Het 1.’ For plotting LD decay curves, the average *r*^*2*^ value (average squared correlations of allele frequencies) was calculated for pairwise markers in 500-kb windows and averaged across the whole genome; this was repeated 10 times. The LD decay rate was measured as the distance at which *r*^*2*^ dropped to half its maximum value.

### Demographic history and population admixture

To better understand historical changes in ancestral TRD and Tule elk population size, demographic history was analyzed with SNP data by applying the pairwise sequentially Markovian coalescent (PSMC) model [46]. This method infers ancestral effective population sizes (*N*_e_) over time, based on the SNP distribution in an individual diploid genome. Parameters were set at -g 6.3, −u 1.5e-08, −p 4 + 25*2 + 4 + 6, −b 100, −× 10,000, −X 10000000, including the average mutation rate (μ) of 1.5 × 10^− 8^ per base per generation in Milu [5] and a generation time (g) of 6.3 years [36].

The demographic history was reconstructed and the pattern of gene flow was elucidated for the TRD and Tule elk populations subsequent to their divergence by using the diffusion approximation for demographic inference (∂a∂i) approach [47], which infers demographic parameters based on a diffusion approximation to the site frequency spectrum. Because reduction in genetic variation in the sex chromosomes will affect estimates of the population genetic parameters, and to ensure selective neutrality, SNPs located on the sex chromosomes were eliminated, and only autosomal SNPs were used for the two analyses of demographic history.

### Genome-wide selective sweep test

To identify genome-wide selective sweeps associated with adaptation to arid environments, the genome-wide distribution of fixation index (*F*_ST_) values [48] and *θ*_π_ ratios were calculated for the TRD and Tule elk populations. The *F*_ST_ values for sliding windows were also calculated using VCFtools [49], for windows 40,000 bp wide sliding in 20,000 bp steps. The *F*_ST_ values were Z-transformed as follows: Z(*F*_ST_) = (*F*_ST_ - μF_ST_) / σ*F*_ST_, where μ*F*_ST_ is the mean *F*_ST_ and σ*F*_ST_ is the standard deviation of *F*_ST_. The *θ*_π_ ratios were log_2_–transformed (π_Tule elk population (control group)_/π_TRD population (arid-desert environment group)_). We then estimated and ranked the empirical percentiles of Z(*F*_ST_) and log_2_ (*θ*_π_ ratio) in each window and considered the windows with the top 5% Z(*F*_ST_) and log_2_ (*θ*_π_ ratio) values simultaneously as candidate outliers under strong selective sweeps. All outlier windows were assigned to corresponding SNPs and genes.

Since the cross-population extended haplotype homozygosity statistic (XP-EHH [50];) maintains power for sample sizes as low as ten [51], XP-EHH was estimated for the TRD and Tule elk populations with XP-EHH version 2012-12-12 (http://hgdp.uchicago.edu/Software/), using windows 10,000 bp wide sliding in 5000 bp steps. The genetic map was assumed to be 2.53 cM/Mb for the CerEla1.0 deer genome [41]. Z scores were calculated using a burn-in of 100 and 1000 sweeps. The threshold for identifying candidate genes in the XP-EHH analyses was set to the top 5% of outlier regions.

We compared the Z(*F*_ST_), log_2_ (*θ*_π_ ratio), and Ztrans (XP-EHH) values of the selective genomic regions with those at the whole-genome scale for TRD and Tule elk. The CerEla1.0 deer reference genome assembly was used to identify the coordinates of nucleotide sequences. Functional information for each gene was obtained by using Blastp to compare the gene set against protein databases, including SwissProt (http://www.uniprot.org), TrEMBL (http://www.uniprot.org/), KEGG (http://www.genome.jp/kegg/), and InterProscan (https://www.ebi.ac.uk/interpro/). As enrichment analyses, the Gene Ontology (GO) term and functional pathway were analyzed for the candidate genes, i.e., all of the annotated genes in the outlier windows with the top 5% Z(*F*_ST_), log_2_ (*θ*_π_ ratio), and Ztrans (XP-EHH) values, using the Go-seq package in R. Specifically, the genes were compared to this classification for functional enrichment analysis of GO biological processes, molecular function, and cellular component terminologies and pathways. Only significantly (*p* < 0.05) over-represented GO terms were considered to be biologically meaningful. The candidate genes were also positioned on known Kyoto Encyclopedia of Genes and Genomes (KEGG) pathways (http://www.kegg.jp) by using KOBAS (KEGG Orthology Based Annotation System [52], selecting cow (*Bos taurus*) genome as orthology.

## Results

### Genome sequencing and mapping

By re-sequencing the whole genomes of 13 TRD individuals at an average depth of 8.57X, we generated 328.167 Gb of raw sequences, which yielded 327.803 Gb of high-quality sequences (Additional file 3: Table S3). We retrieved 113.394 Gb of sequence for the Tule elk from the NCBI Sequence Read Archive (SRA; BioProject ID PRJNA345218), with 88% of the data showing Phred quality scores higher than 30 and the aligned high-quality genomic data at an overall effective sequence depth of 40 for subsequent analyses [34]. Based on the high-quality sequence data, the genomic GC content averaged 44.03% for the TRD (Additional file 3: Table S3) and 41.55% for the Tule elk [34], which are typical values comparable to those observed in the genomes of other mammals, including ungulates [25, 28–30], indicating that our sequencing data were not affected by GC bias. The mapping rates of our samples to the CerEla1.0 reference genome assembly ranged from 88.77 to 90.35% (Additional file 4: Table S4).

We identified 8,520,805 SNPs after quality control for TRD and 4,896,658 for Tule elk. Most of the high-quality SNPs were located in intergenic (7,002,807) and intronic regions (1,321,254), with only 36,192 synonymous and 27,623 nonsynonymous SNPs located within exons in TRD (Additional file 5: Table S5). Among the SNPs of TRD, heterozygous SNPs for individuals ranged from 1,603,886 to 2,184,617 (Additional file 6: Table S6). As a measure of the quality of our SNP data, the transition-transversion (ts/tv) ratio for TRD and Tule elk was 2.64 and 2.63, respectively, which was consistent with the extensive analysis of mammalian genes by Li [53].

### Demographic history

**Fig. 2:**
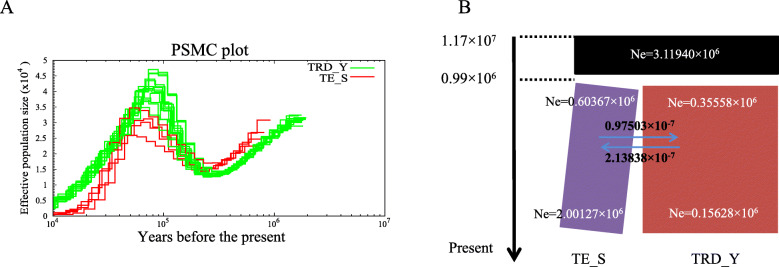
Population histories of Tarim red deer (TRD_Y) and Tule elk (TE_S) populations. **a** PSMC analysis inferring variation in *N*_*e*_ over the last 10^6^ years for the Tarim red deer and Tule elk populations. Generation time (g) = 6.3 years, and neutral mutation rate per generation (*μ*) = 1.5 × 10 ^− 8^. **b** ∂a∂i analysis showing effective population sizes for the ancestral population and the Tarim red deer and Tule elk populations from ~ 1.17 × 10 ^7^ years ago to the present. The average number of migrants per year between the two populations in each time interval is indicated by the labeled arrows

Genome-wide patterns of heterozygosity provide useful information on long-term effective population sizes. We reconstructed historical fluctuations in *N*_e_ for TRD and Tule elk with the PSMC model and identified two bottlenecks and one expansion the two populations, which showed highly similar patterns of historical fluctuation in *N*e (Fig. 2a). The first continuous decline in *N*_e_ was estimated to have taken place between 1.8 and 0.25 Mya, after which the ancestral TRD population expanded until about 0.075 Mya (Fig. 2a), followed by another decline in *N*_e_. The ∂a∂i model, on the other hand, revealed that the TRD and Tule elk populations diverged ~ 0.98 Mya, at an estimated *N*_e_ of 0.356 × 10^6^ for TRD and 0.604 × 10^6^ for Tule elk (Fig. 2b, Additional file 7: Table S7). The present *N*_e_ for TRD was 0.156 × 10^6^, an apparent decline of more than two-fold since its initial divergence. Very low levels of gene flow were calculated between TRD and Tule elk (2.13 × 10^− 7^ migrants per year from TRD to Tule elk; 0.975 × 10^− 7^ from Tule elk to TRD), which accords with their geographic distributions and divergence time [8, 13, 54].

### Patterns of genomic variation and linkage disequilibrium

**Fig. 3:**
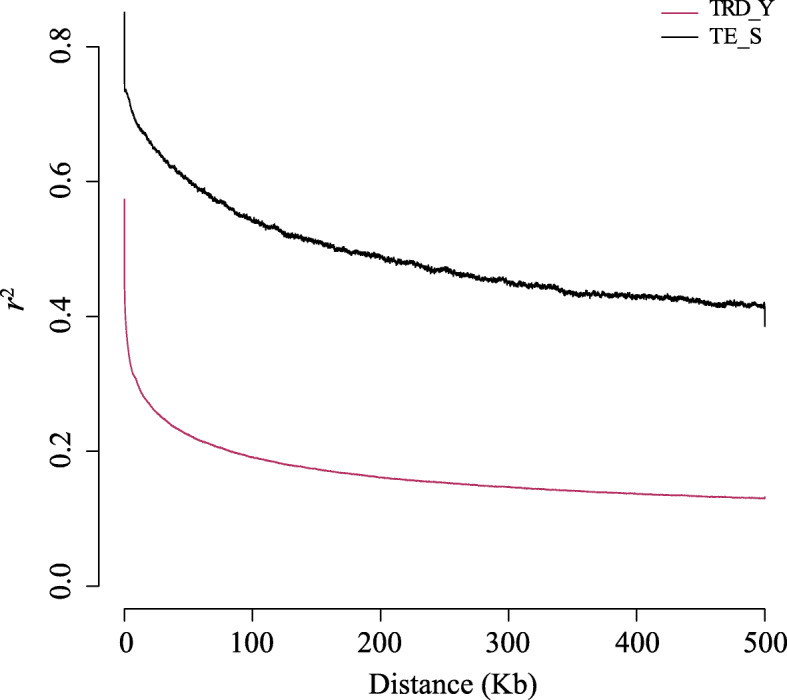
Linkage disequilibrium (LD) patterns for the Tarim red deer (TRD-Y) and Tule elk (TE-S) populations

The genome-wide average *θ*_*π*_ value was 0.561 × 10^− 3^ for TRD and 0.240 × 10^− 3^ for Tule elk. The Tule elk population exhibited an overall slow decay rate and a high level of linkage disequilibrium (LD), whereas the TRD population showed a rapid decay rate and a low level of LD (Fig. 3). For the whole genomes, the LD decay rate was estimated at ~ 437.1 kb for Tule elk and ~ 13 kb for TRD, with *r*^*2*^ dropping differentially to 0.4257 and 0.2870, respectively. These differences in the genome-wide LD patterns suggest that natural selection differentially affected the population size, structure and history between the two populations, as LD is a comprehensive reflection of various evolutionary forces.

### Selective sweep and GO enrichment analyses

**Fig. 4:**
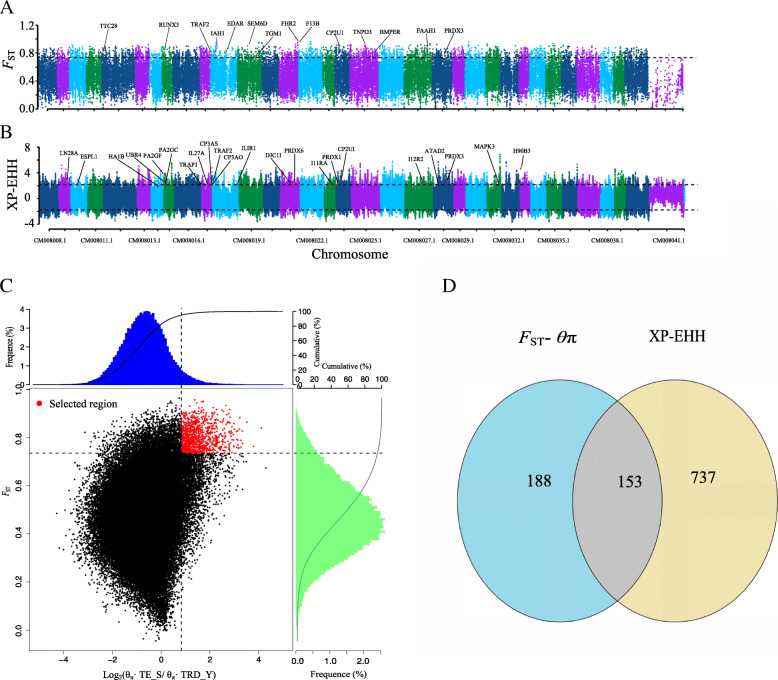
Genomic regions with strong selective signals in Tarim red deer. **a** Distribution of *F*_ST_ values calculated in 10-kb sliding windows. **b** Distribution of XP-EHH values calculated in 10-kb sliding windows. The dotted line above the figure corresponds to the top 5% of outliers with XP-EHH values > 2.1376. **c** Distribution of log_2_ (*θ*π•control/*θ*π•Tarim red deer) and the top 5% highest Z(*F*_ST_) values calculated in 40-kb sliding windows with 20-kb increments between Tarim red deer and the control Tule elk population. Data points in red [corresponding to the top 5% of the empirical log_2_ (*θ*π ratio) with values > 0.827 and the top 5% of the empirical Z(*F*_ST_) distribution with values > 0.734] are genomic regions under selection in Tarim red deer. The genes visualized in (**a**) and (**b**) are candidate genes in the Tarim red deer. **d** Venn diagram of candidate genes screened by *F*_ST_-*θ*π and XP-EHH in Tarim red deer

We considered the top 5% of *F*_ST_ values and the *θ*_*π*_ ratio cut-off values, Z(*F*_ST_) > 0.734 and log_2_ (*θ*_*π*_ ratio) > 0.827 (Fig. 4c) for the Tule elk population (control group) / TRD population (arid-desert environment group), to be potentially selected regions associated with adaptation to an arid-desert environment. We identified 955 putative regions spread across all chromosomes, comprising of 341 candidate genes (Fig. 4a, Additional file 8: Table S8). Among these genes, however, nine did not match any annotated genes, due either to incomplete annotation of the genomes used in this study, or more likely to the selected functional mutations within each of these regions not being located within or close to a protein-coding gene [24]. In addition, the XP-EHH analysis identified 890 genes (top 5% outliers, XP-EHH value > 2.1376) as positive candidates for involvement in adaptation to an arid-desert environment (Fig. 4b, Additional file 9: Table S9). Overall, using multiple approaches, we identified 153 overlapping candidate genes for adaptation to an arid-desert environment (Fig. 4d, Additional file 10: Table S10).

**Fig. 5:**
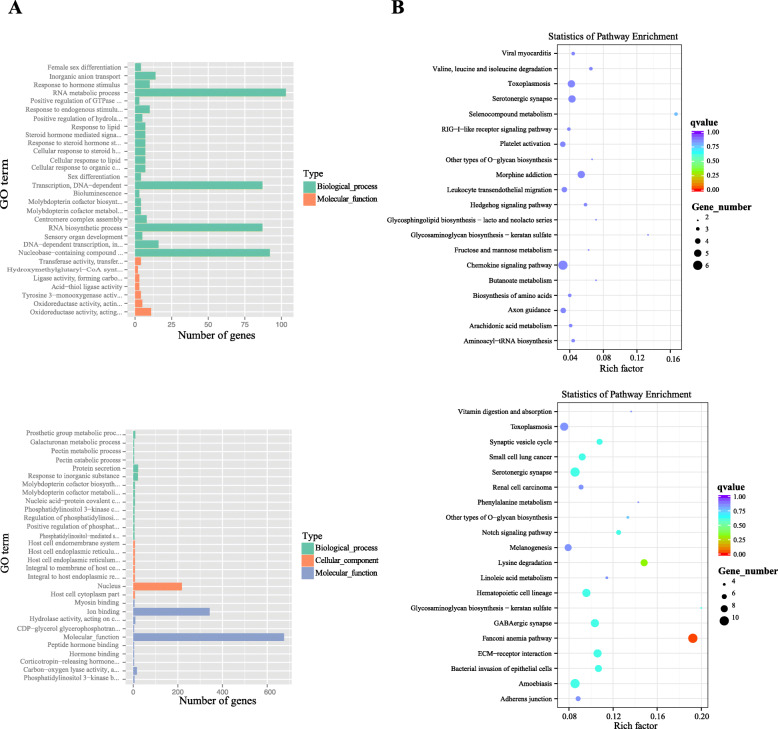
Enriched GO terms (**a**) and KEGG pathways (**b**) for genes detected in the selective sweep *F*_ST_-*θ*π ratio (above) and XP-EHH (lower) analyses

The candidate genes identified by *F*_ST_ - *θ*_*π*_ method were enriched in various fundamental biological processes and molecular functions, and were highlighted by 90 significant gene ontology (GO) terms (*p*<0.05, without Bonferroni correction) (Fig. 5a, Additional file 11: Table S11). The top several categories from the GO database associated with these genes included transferase activity, inorganic anion transport, cellular response to stimulus, metabolic and biosynthetic process (such as RNA metabolic and biosynthetic process, transcription), oxidoreductase activity, hydrolase activity, and regulation of signaling pathway (Additional file 11: Table S11).

We also performed a gene-set enrichment test for KEGG pathways and GO categories for the genes selected by the XP-EHH method and identified significantly enriched categories. Most of the candidate genes were associated with multiple signaling, signal transduction pathways, and various metabolic pathways (Fig. 5). Enrichment tests revealed GO categories that were significantly related to myosin binding, prosthetic group metabolic process, protein secretion, ion binding, galacturonan metabolic process, pectin metabolic-catabolic process, response to inorganic substance, regulation of immune system process, response to water stimulus, and sensory organ development (Additional file 12: Table S12). Among the top KEGG pathways, the candidate genes were significantly enriched in fanconi anemia pathway, lysine degradation, ECM-receptor interaction, Notch signaling pathway, bacterial invasion of epithelial cells, Linoleic acid metabolism, melanogenesis, and others (Additional file 13: Table S13).

## Discussion

### Demographic history

The first period of decline in *N*_e_ (Fig. 2a) for TRD coincided with a shift in the climate cycle 1–0.25 Mya [55]. Decreases in *N*_e_ have also been reported for the giant panda (*Ailuropoda melanoleuca* [56];), brown bear (*Ursus arctos* [57];), and snub-nosed monkeys (*Rhinopithecus roxellana*, *R. brelichi* and *R. bieti* [58]) during this period, when global glaciation, cold climate, and frequent fluctuations in sea level [59] had a profound evolutionary effect on the population sizes of some mammalian species.

The timing of ancestral population expansion for TRD (Fig. 2a) coincided with the end of the interglacial period (0.13–0.07 Mya [60];), when environmental conditions became similar to those at present [61]. A recovery of *N*_e_ occurred in *R. roxellana* around the same time (~ 0.07 Mya [58];), but that for giant panda was inferred to have occurred later, around 30,000–40,000 years ago [56]. The population expansion in TRD was likely due to the relatively warm, wet climatic conditions during the last interglacial period, allowing organisms to recolonize the northeast Tarim as glacial melt-water increased and rivers resumed their courses [62]. TRD underwent a subsequent decline in *N*_e_ after 0.075 Mya. This time interval coincided with extreme cooling during the last glaciation [59], when the climate became colder and drier, and the meltwater that served as the main source of water in the Tarim Basin greatly decreased [63]. The northern and eastern parts of the Tarim River dried up, and the resident biota must have either gone locally extinct or retreated to the southwest [62].

On the other hand, the latter decrease in TRD *N*_e_ coincided with the dispersal of modern humans from Africa to Eurasia [64] which caused a similar decrease in *N*_e_ in several other mammals [56–58, 65]. Lorenzen et al. [59] suggested that the decline in genetic diversity observed in horse and bison after the last glaciation, and to a lesser extent in reindeer, might reflect the impact of expanding human populations in Europe and Asia. We similarly observed reduced genomic diversity in TRD relative to other mammals (Additional file 14: Table S14). We thus hypothesize that the combination of an extremely cold climate and anthropogenic effects is responsible for the second reduction in the TRD *N*_e_.

Interestingly, the divergence time of ~ 0.98 Mya between TRD and Tule elk (Fig. 2b, Additional file 7: Table S7) estimated by using the ∂a∂i model differed from that estimated by mitochondrial Cyt *b* and D-loop data [66], in which the most recent common ancestor between Eastern (*C. canadensis* and *C. nippon*) and Western (*C. elaphus* and *C. hanglu*) Clades may have occurred in the late Miocene, at a mean age of approximately 6 Mya, but was consistent with an estimate based on genomic SNP data that the isolation of TRD and *C. canadensis* occurred 0.8–2.2 Mya [67], suggesting that TRD colonized the Tarim Basin more recently than previously thought [66].

### Patterns of genomic variation and linkage disequilibrium

In agreement with previous studies based on single or multiple genes [37, 68–70], *θ*_π_ values for the TRD and Tule elk populations (0.561 × 10^− 3^ and 0.240 × 10^− 3^, respectively) showed a lower level of genomic diversity than that of other mammals [28, 30, 71, 72], and was similar to or even lower than that of some endangered mammals (Additional file 14: Table S14). Most of these species have gone through bottleneck events at least once in their evolutionary history, suggesting that historical changes in population size contributed to the loss of genetic variation (e.g. [73, 74]), including that in TRD. Corroborating this conclusion, Broughton et al. [38] provided provisional evidence, based on DNA from archaeological elk bones, that a decline in genetic diversity in the California Tule elk (included in our study) was due to a historical bottleneck event.

Other factors could also have contributed to the low genomic diversity to some extent, such increased human activities and the degraded and fragmented habitat of the TRD population [75, 76]. Higher LD in the Tule elk population (Fig. 3) may have contributed to reduced genomic variation, since selection in cases of relatively high LD can reduce neutral nucleotide diversity by strong selection against deleterious alleles or by substitutions of beneficial alleles at linked loci [77–79]. Finally, genetic variation responds to experimental manipulations of population size [80, 81], and we cannot rule out that low genetic variation in TRD and Tule elk might be due to small population sizes and/or inbreeding effects [75, 82].

### Adaptive signatures to an arid-desert environment

The Z(*F*_ST_) and log_2_ (*θ*_π_ ratio) values we estimated for selected genomic regions of TRD from an arid-desert zone were much higher than the overall genomic level (Additional file 15: Fig. S1). Relative to the overlap expected by chance, we observed a marked excess of overlapping selective signals shared between the candidate genes identified in this study and candidate genes published previously for other mammals in the same or a similar environment (Additional file 16: Fig. S2). Likewise, a number of the candidate genes overlapped among the *F*_ST_, *θ*_π_, and XP-EHH methods (Fig. 4d, Additional file 10: Table S10). Hence, it is evident that the candidate genes identified in this study were reliable.

Several overlapping selective signals were shared between the candidate genes identified by the *F*_ST_-*θ*_π_ ratio here and the predefined gene panel for previously published candidate genes in other mammalian species in a similarly extreme environment (Additional file 16: Fig. S2). For example, several genes with high selective sweeps in TRD have also been identified as candidate genes in the adaptation of Bactrian camels (*TRAF2, PRDX3, EDAR, TGM1, F13B, IAH1*) and sheep breeds (*RUNX3, TNPO3* and *TTC28*) to desert environments [25, 28]. *EDAR* (Tumor necrosis factor receptor superfamily member EDAR) functions in the repair of skin tissue damage, maintains skin microenvironment homeostasis [83], and is involved in the formation and regulation of hair curl, which helps in temperature homeostasis and protects against heat damage [84]. Furthermore, Bornert et al. [85] found that the more the function of mouse *EDAR* was preserved, the shorter the mandible was. This parallels the significantly shorter and wider skull morphology of TRD compared to congeners like the Tianshan red deer (*C. e. songaricus*) and Altai red deer (*C. e. sibiricus*) in Xinjiang, China; this morphology is presumably adaptive to eating food hardened by dryness and heat [86]. We hypothesize that the *EDAR* gene in TRD is involved in the development of hair and sweat glands, which likely play important roles in regulating body temperature and maintaining water-salt balance, and is related to adaptive skull morphology, but these functions need to be confirmed.

The functions of the GO terms for candidate genes selected by *F*_ST_-*θ*_π_ ratio were quite consistent with adaptive features of TRD, such as the relatively compact, medium-sized body and high salt-alkali tolerance. Our results agree well with the conclusions of Yang et al. [25] that the nervous system development, metabolic process, and response to stimulus terms are related to adaptations of sheep breeds to desert environments. The KEGG pathway analysis indicated that these selected genes were enriched in several biological pathways, including selenocompound metabolism, toxoplasmosis, the hedgehog signaling pathway involved in ovarian activity [87], valine, leucine and isoleucine degradation, and others (Fig. 5, Additional file 17: Table S15).

Some candidate genes (Additional file 16: Fig. S2) in TRD located in the selective sweep regions identified by the XP-EHH analysis were related to the adaptation of Bactrian camels to a desert environment (*TRAF2, UBR5, DBX1, PRDX3, SLX4, TPO* and *NLE1* [28];), and sheep breeds to the Taklimakan Desert (*EHBP1, TTC28, IRF5, RNF24, TSHB* and *KCP* [25];) and to arid environments (*CCL22, RUNX3, STK40, HECW2, NDOR1 ATAD2* and *SMC1B* [25];). TRD and sheep breeds inhabit same environment in the Tarim Basin, and overlapping candidate genes indicate that species in the basin have faced similar selective pressures and have undergone convergent evolution. Apart from these candidate genes, none of our candidate regions overlapped with those identified in previous studies, perhaps due to different study designs, sample sizes, sequencing coverage, and statistical methods, as well as species-specific differences. We found 16 candidate genes (*TGM1*, *FHR2*, *RPN2*, *GPR98*, *PK3CB*, *I12R2*, *IL1R1*, *TBA*, *CABP8*, *TCPA*, *VINC*, *CASP3*, *LAMB2*, *LAMB1*, *ITAM* and *CO4A4*) were also identified by the XP-EHH analysis to be significantly over-represented in GO categories and KEGG pathways (Additional file 12: Table S12 and Additional file 13: Table S13), including genes related to myosin binding (GO:0017022, *p* < 0.01), regulation of immune system process (GO:0002682, *p* < 0.05) and immune response (GO:0050776, GO:0050778, *p* < 0.05), bacterial invasion of epithelial cells (bta05100, *p* < 0.05), ECM-receptor interaction (bta04512, *p* < 0.05), amoebiasis (bta05146, *p* < 0.05), small cell lung cancer (bta05222, *p* < 0.05), renal cell carcinoma (bta05211), toxoplasmosis (bta05145), HTLV-I infection (bta05166), NF-kappa B signaling pathway (bta04064), and apoptosis (bta04210). These clusters and pathways are immunologically relevant to adaptation to arid-desert conditions, because many of them are involved in disease and pathogen resistance. TRD suffer diseases such as tuberculosis [88, 89], pasteurellosis [90], and cysticercustenuicollis [91]; in addition, parasites like hard ticks, widely distributed in the Gobi Desert within the Tarim Basin, are a main threat during the hot season [92]. It is thus plausible that genes related to the immune system would be targeted by natural selection in TRD. Previous studies for other mammalian species have similarly reported genes related to the immune system to be involved in adaptation to hot, arid environments [3, 93].

The Tarim Basin has high solar radiation, and long-term exposure to ultraviolet radiation can lead to a number of ophthalmic conditions in animals. Five candidate genes (*LAMB1*, *LAMB2*, *CYC*, *FANCF* and *GPR98*) were positively selected by XP-EHH analysis in TRD are functionally involved in ocular development, visual protection, and photoreceptor cell synapses [94–96]. Two of these (*CYC* and *FANCF*) evolved more rapidly in the Bactrian camel and dromedary exposed to long-term ultraviolet radiation than in the alpaca [28]. These genes may play a central role in visual perception in TRD, and selection on eye physiology may be an important feature of adaptation to arid-desert conditions.

Relevant to the adaptation of TRD to arid conditions, ten selected genes (including *SLX4*, *FANCF*, *FANCG*, *FANCI*, *ATR*, and *POLH*) were related to the fanconi anemia pathway (bta03460*, p* < 0.01) (Additional file 13: Table S13) involved in DNA repair, DNA replication fork stability, and other cellular processes [97]. In addition, the peroxiredoxins (*PRDX1*, *PRDX3* and *PRDX6*) we identified in the high selective sweep regions belong to a family of peroxidases that regulate the level of reactive oxygen species (ROS) in cells, are involved in antioxidant protection and cell signaling, and are associated with increased resistance to radiation [98]. *PRDX3* was enriched in the oxidoreductase activity (GO: 0016628, GO: 0016627, *p* < 0.05,) and response to various stimulus (GO: 0009725, GO: 0009719, GO: 0033993, GO: 0048545, GO: 0071383, *p* < 0.05, Additional file 11: Table S11) GO categories. These candidate genes are likely important for the survival of TRD in an arid environment by playing significant roles in prevention of DNA damage, DNA repair, oxidative stress, and stress responses.

The TRD inhabits the edge of Taklimakan Desert, where sandstorms are frequent and airborne dust can initiate respiratory diseases such as asthma. The candidate gene *TRAF2* (TNF receptor–associated factor 2 protein) was identified for TRD in both the *F*_ST_-*θ*_π_ and XP-EHH approaches was also a critical gene in the adaptation of Bactrian camels to a desert environment [28]. Involved in the signal transduction of immune function related receptors (e.g. CD40, CD30, CD27, 4-1BB and RANK) and combined with RIP, GCK, ASKl and NIK, *TRAF2* plays an important role in the regulation of most kinases and their corresponding transcription factors [99, 100]. In addition, high level of *TRAF2* expression in asthmatic rats and humans showed that it is more likely involved in the inflammatory mechanism of asthma possibly through airway epithelial cells and inflammatory cells [101]. Meanwhile, *IL1R1* (Interleukin-1 receptor type 1)*,* positively identified under XP-EHH selection in TRD, is the signal-transducing receptor for IL-1 and encodes cytokine receptor that participate in host defense mechanisms, including immune and inflammatory responses [102]. Playing a crucial role in IL-1 signaling pathway, *IL1R1*can increase the effect of IL-1, which is critically involved in the regulation of inflammation and pathobiology of immune and inflammatory conditions, such as asthma and allergic diseases [103, 104]. We thus speculate that *TRAF2* and *IL1R1* may help defend against airborne dusts in TDR, and that selection on respiratory physiology helps TRD adapt to its arid-desert environment.

Among the candidate genes for arid-desert adaptation we obtained by XP-EHH analysis, *CP2U1*, *PGH1*, *PTGDS*, *HYES*, *PA2GF* and *PA2GC* are functionally involved in the arachidonic acid metabolic pathway (bta00590, Additional file 13: Table S13), which was likewise identified as highly functionally important in desert adaptation in the Bactrian camel [27] and sheep breeds [25], indicating convergent evolution. Four candidate genes (*PA2GF*, *PA2GC*, *CP3AS* and *CP3AO*) are involved in linoleic acid metabolism (bta00591, Additional file 13: Table S13), where *PA2GF* and *PA2GC* play a vital role in converting lecithin into linoleic acid. *CP2U1* (cytochrome P450 2 U1), *CP3AS* (cytochrome P450 3A28), and *CP3AO* (cytochrome P450 3A24) belong to the cytochrome P450 (CYP) family, members of which display a wide variety of functions in the metabolism of many xenobiotic species, including the endogenous biosynthesis of fatty acids, bile acids, steroids, or vitamins [105, 106]. The *CP2U1* (bta00590, Additional file 13: Table S13) gene product, in particular, can help to transform arachidonic acid into 19(S)-HETE and 20-HETE. 19(S)-HETE is a potent vasodilator of the renal preglomerular vessels that stimulate water reabsorption [107], and is potentially useful for the survival of camels in the desert [27]. 20-HETE is a potent vasoconstrictor in the kidney and brain and is involved in the pathogenesis of hypertension [108, 109], which is consistent with the consumption of highly mineralized water and salty foods (i.e. *Phragmites communis* and *Tamarix ramosissima*) by TRD [5, 13, 110]. It appears, then, that arachidonic acid, linoleic acid, and fatty acid metabolisms, and particularly the *CP2U1* gene, are important factors for the survival of TRD in its arid environment. As in Onzima et al. [26], the candidate gene *MAPK3* (mitogen-activated protein kinase 3) may also play role in desert adaptation; this gene is related to encoding the Na^+^/K^+^-ATPase and the epithelial Na^+^ channel and is thus involved in the reabsorption of sodium in the kidney [28].

The candidate genes *H90B3* (putative heat shock protein HSP 90-beta-3, known as *HSP90AB3P* in humans) and *TRAP1* (mitochondrial heat shock protein 75 kDa) also identified by XP-EHH analysis are involved in ion binding (GO:0043167, *p* < 0.01), transition metal ion binding (GO: 0046914, *p* < 0.05), oxidoreductase activity (GO: 0016652, *p* < 0.05), and metabolic process (GO:0008152, *p* < 0.05) (Additional file 12: Table S12), which may be related to heat stress response [111]. *DJC11* (DNAJ homolog subfamily C member 11, known as *DNAJC11* in humans) and *AUXI* (putative tyrosine-protein phosphatase auxilin, known as *DNAJC6* in *Bos taurus*) are associated with the heat shock family of proteins [112]. The identification of multiple genes associated with heat stress suggests that these genes are involved in the adaptation TRD to a hot environment. Moreover, we found enrichment for eight candidate genes (*GNAI2*, *FZD4*, *MP2K2*, *CREB3*, *CBP*, *GNAO*, *TF7L2* and *GNAO*) in the melanogenesis pathway (bta04916, Additional file 13: Table S13). This is consistent with the dark gray-brown coat color of TRD in summer turning to light gray-brown in winter, and the obvious black-brown back line. Melanogenesis associated with heat-tolerance appears to be common among species adapted to desert and desert-like environments [3]. Accordingly, we assumed that this KEGG pathway is likely involved in the coat color and thermal tolerance traits of this species.

We found intriguing over-represented biological pathways based on raw *p*-values, though the results of the more stringent Bonferroni correction were not statistically significant. This is likely attributable to the small sample size and relatively low coverage of sequencing depth in this study and may also due to the genomic differences between the red deer and the Tule elk. However, our results provide a useful indication of the mechanisms involved in arid-desert adaptation in TRD. We found putative signatures containing a complex of genes, pathways, and GO terms directly or indirectly related to oxidative stress, water reabsorption, immune-regulation, energy metabolism, eye protection, heat stress, the respiratory system, prevention of high blood pressure, and prevention and repair of DNA damage. As with previous studies [3, 25, 28, 113], our study underscores that arid-desert adaptation is complex, involving various biological processes and quantitative trait loci, each contributing a small but cumulative effect to the overall phenotype.

## Conclusions

The genomic sequencing data for TRD could be valuable and useful for both conservations of wild populations and utilization of domestic ones as a resource for further study, providing baseline sequence and annotation data for the species. Meanwhile the selective sweep results advance our understanding of the genetic mechanisms underlying the adaptations of TRD and other species in similar extreme environments. However, as the samples we analyzed were minimal and to some extent the Tule elk we use as comparison was somehow distantly related to Tarim red deer, more detailed studies are necessary to further confirm and refine our results by integrating comprehensive genomic data (for example, candidate gene sequencing, high density SNP genotyping, gene expression profiling) with environmental and physiological data, and to identify underlying genomic mechanisms.

## Supplementary information

**Additional file 1: Table S1.** Identity, location, and sample type for *Cervus elaphus yarkandensis* and *Cervus canadensis nannodes* samples used in this study.

**Additional file 2: Table S2.** The explanation of parameters in samtools ‘mpileup’ command used for SNP calling.

**Additional file 3: Table S3.** Summary of sequence data generated in this study.

**Additional file 4: Table S4.** Statistics for mapping rate and coverage of samples analyzed in this study.

**Additional file 5: Table S5.** SNP annotation statistics of Tarim red deer and Tule elk.

**Additional file 6: Table S6.** Individual SNP statistics of Tarim red deer.

**Additional file 7: Table S7.** Parameters inferred from the diffusion approximation for demographic inference (∂a∂i) approach.

**Additional file 8: Table S8.** List of genes in the overlapping regions selected by the top 5% highest log_2_(*θπ*•control/*θπ*•Tarim red deer) and top 5% highest Z(*F*_ST_) scores for Tarim red deer.

**Additional file 9: Table S9.** List of genes in the overlapping regions selected by the top 5% highest XP-EHH values for Tarim red deer in XP-EHH analyses.

**Additional file 10: Table S10.** List of genes in the selected overlapping regions by top 5% highest log_2_ (*θ*_π•control_/*θ*_π•Tarim red deer_) and top 5% highest Z (*F*_ST_) for Tarim red deer as well as the significance of these genes in XP-EHH (XP-EHH value) analyses.

**Additional file 11: Table S11.** GO functional enrichment of the candidate genes obtained by *F*_ST_- *θ*π ratio analysis that are associated with adaptation to arid environments.

**Additional file 12: Table S12.** GO functional enrichment of the candidate genes obtained by XP-EHH analysis associated with the drought environment adaptations.

**Additional file 13: Table S13.** The top 30 KEGG pathway enrichment of the candidate genes obtained by XP-EHH analysis associated with the drought environment adaptations.

**Additional file 14: Table S14.** Summary statistics for genomic nucleotide diversity in different mammalian species.

**Additional file 15: Figure S1.** Differences in Z(*F*_ST_) and *θ*π ratio values between the selected regions and the whole-genome scale for Tarim red deer (TRD). (A) Comparison between Z(*F*_ST_) values for genomic regions that have undergone selective sweeps and Z(*F*_ST_) values at the whole-genome scale in TRD. The upper, middle (within the box), and lower boundary lines for the boxes represent 25, 50% (median value), and 75% of the Z(*F*_ST_) and *θ*π ratio values, respectively. (B) Comparison between *θ*π ratio values for genomic regions that have undergone selective sweeps and *θ*π ratio values at the whole-genome scale.

**Additional file 16: Figure S2.** Venn diagrams showing the overlap of candidate genes identified by the *F*_ST_ & *θ*π (A) and XP-EHH (B) analyses in Tarim red deer. Numbers in the intersecting regions are the observed overlapping genes among the candidate genes in Tarim red deer, and predefined gene panel, i.e., previously published candidate genes in other mammalian species in arid environments, including the Bactrian camel, sheep breeds from the Taklimakan Desert region, and sheep breeds from arid regions.

**Additional file 17: Table S15.** Top 30 KEGG pathway enrichment of the candidate genes obtained by *F*_ST_- *θ*π ratio analysis associated with the drought environment adaptations.

## Data Availability

The datasets analyzed during the current study are available from the corresponding author on reasonable request.
