# Supplementary material for: Whole-genome sequencing of Tarim red deer (Cervus elaphus yarkandensis) reveals demographic history and adaptations to an arid-desert environment
Source: Front Zool. 2020 Oct 16;17:31. doi: 10.1186/s12983-020-00379-5 (PMC7565370; doi:10.1186/s12983-020-00379-5)
Supplement: Supplementary file 15 — Additional file 15: Figure S1. Differences in Z(F ST) and θπ ratio values between the selected regions and the whole-genome scale for Tarim red deer (TRD). (A) Comparison between Z(F ST) values for genomic regions that have undergone selective sweeps and Z(F ST) values at the whole-genome scale in TRD. The upper, middle (within the box), and lower boundary lines for the boxes represent 25, 50% (median value), and 75% of the Z(F ST) and θπ ratio values, respectively. (B) Comparison between θπ ratio values for genomic regions that have undergone selective sweeps and θπ ratio values at the whole-genome scale. [file 12983_2020_379_MOESM15_ESM.pdf]

A

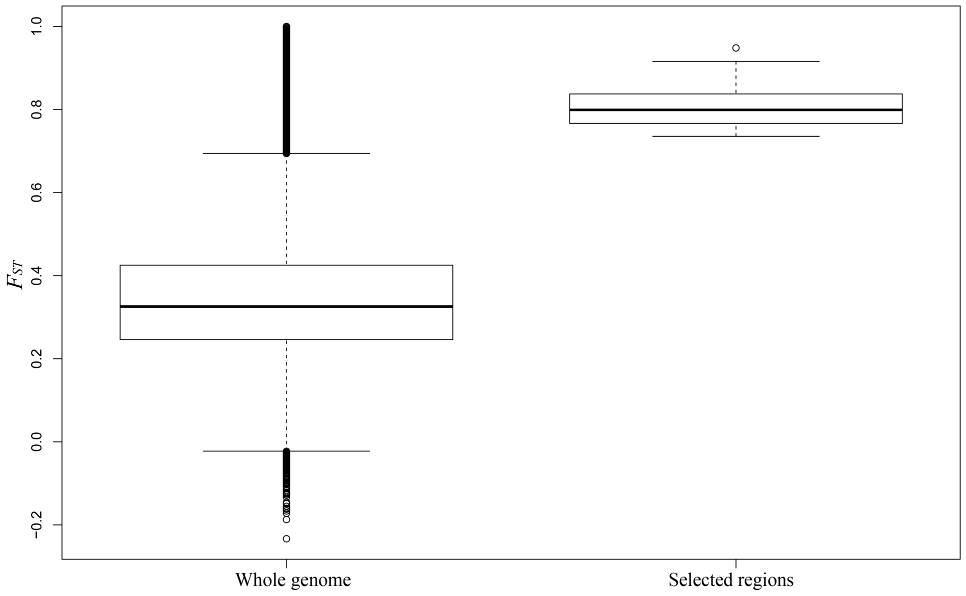

B

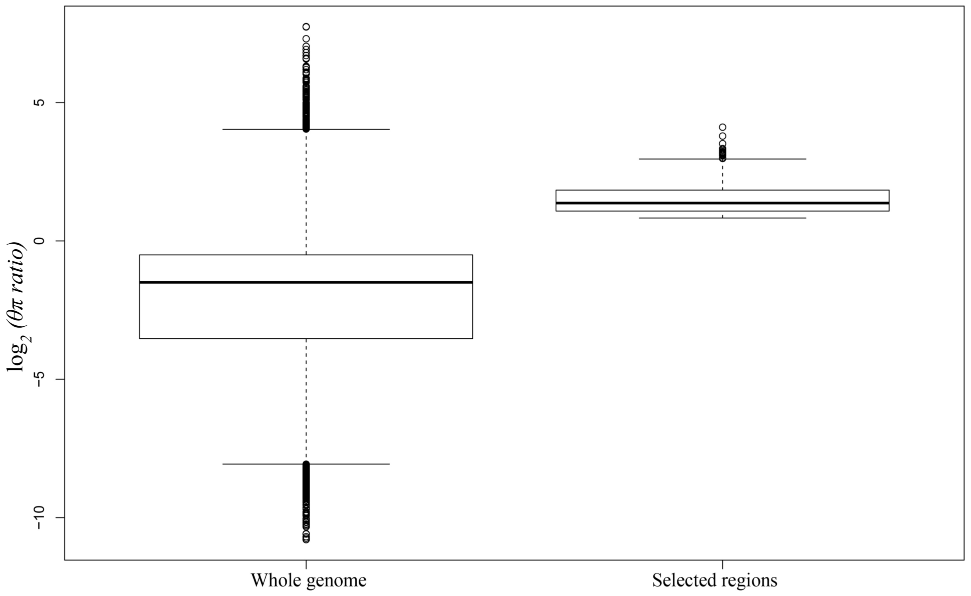

Additional file 15: Fig S1. Differences in $Z(F_{ST})$ and $\theta\pi$ ratio values between the selected regions and the whole-genome scale for Tarim red deer (TRD). (A) Comparison between $Z(F_{ST})$ values for genomic regions that have undergone selective sweeps and $Z(F_{ST})$ values at the whole-genome scale in TRD. The upper, middle (within the box), and lower boundary lines for the boxes represent 25%, 50% (median value), and 75% of the $Z(F_{ST})$ and $\theta\pi$ ratio values, respectively. (B) Comparison between $\theta\pi$ ratio values for genomic regions that have undergone selective sweeps and $\theta\pi$ ratio values at the whole-genome scale.
