# Supplementary material for: Whole-genome sequencing of Tarim red deer (Cervus elaphus yarkandensis) reveals demographic history and adaptations to an arid-desert environment
Source: Front Zool. 2020 Oct 16;17:31. doi: 10.1186/s12983-020-00379-5 (PMC7565370; doi:10.1186/s12983-020-00379-5)
Supplement: Supplementary file 16 — Additional file 16: Figure S2. Venn diagrams showing the overlap of candidate genes identified by the F ST & θπ (A) and XP-EHH (B) analyses in Tarim red deer. Numbers in the intersecting regions are the observed overlapping genes among the candidate genes in Tarim red deer, and predefined gene panel, i.e., previously published candidate genes in other mammalian species in arid environments, including the Bactrian camel, sheep breeds from the Taklimakan Desert region, and sheep breeds from arid regions. [file 12983_2020_379_MOESM16_ESM.pdf]

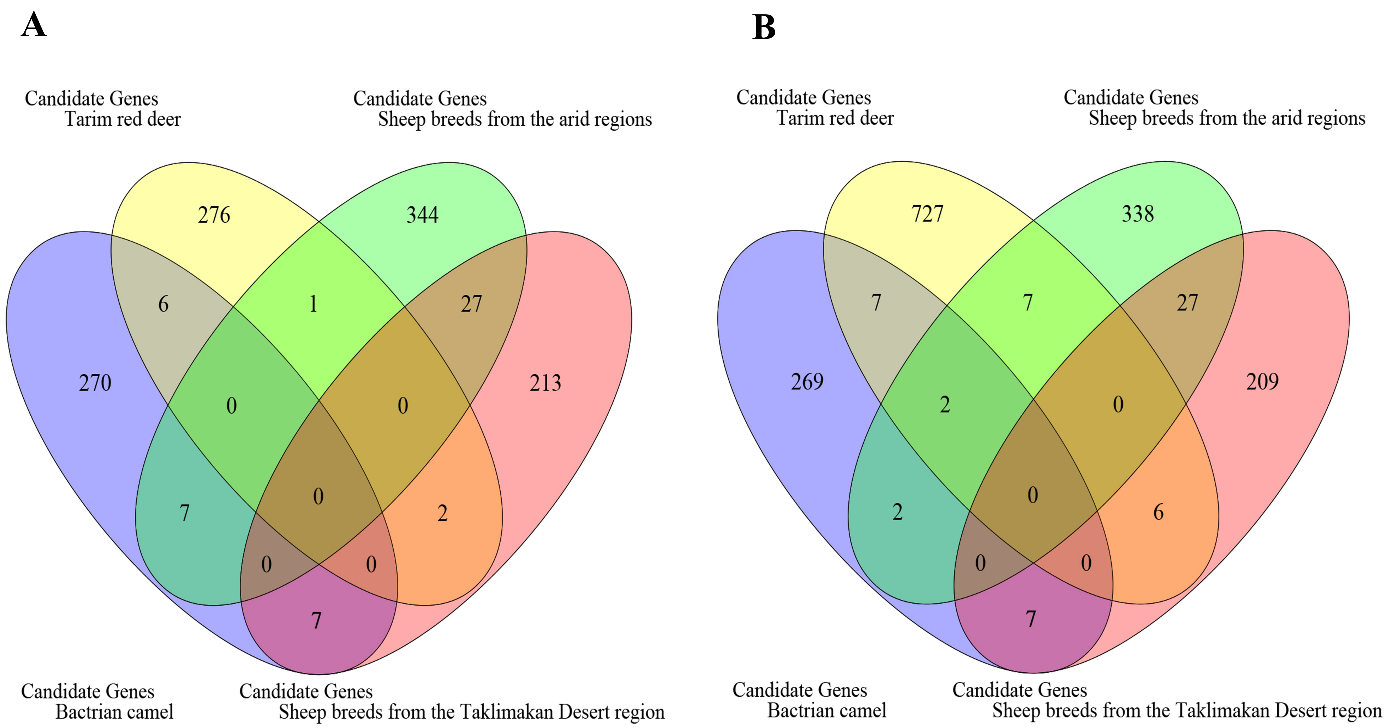

Additional file 16: Fig S2. Venn diagrams showing the overlap of candidate genes identified by the F_{ST} & $\theta\pi$ (A) and XP-EHH (B) analyses in Tarim red deer. Numbers in the intersecting regions are the observed overlapping genes among the candidate genes in Tarim red deer, and predefined gene panel, i.e., previously published candidate genes in other mammalian species in arid environments, including the Bactrian camel, sheep breeds from the Taklimakan Desert region, and sheep breeds from arid regions.
